# Surgery at Buena Vista

**Published:** 1847-10

**Authors:** W. B. Herrick

**Affiliations:** Professor of Anatomy in Rush Medical College, and late Surgeon 1st. Regiment Illinois Volunteers


ARTICLE IV.

*Surgery at Buena Vista.* By W. B. Herrick, M. D., Professor of Anatomy in Rush Medical College, and late Surgeon 1st. Regiment Illinois Volunteers.

Our small army, of about 5000 only, mostly volunteers, under the command of Gen. Taylor, was occupying the position of Agùa Nueva, ten miles from San Louis Potosi, when it began to be reported in camp that Santa Anna, with a force of more than 20,000, was on his march from the latter place to attack us.

Rumors of the enemy's advance had arisen frequently, and caused so many false alarms in camp during the winter, that but little credit was given to this report, until after the return of one of our reconnoitering parties with the report of having actually passed through the enemy's encampment not sixty miles from us, and within one day's forced march of our position.

Early on the morning of the 22d, Santa Anna arrived at Agua Nueva, at which time and place he had evidently intended to attack us. But in this he was disappointed by a prompt movement of our commanding general, who had in the mean time abandoned this comparatively weak position, leaving a few waggons and some stores in order to give the appearance of his having retreated precipitantly, and taken possession of the pass near the place, now so justly celebrated, called Buena Vista.

By dawn of day on the morning of the 22nd, all our pickets had been driven in by the advance of the Mexican army, and by ten o'clock, A. M., the main body could be plainly seen from our position, advancing in dense columns, marked by clouds of dust, extending as far as the eye could reach.

During this time an occasional volley of musketry could be heard, showing that skirmishing had commenced between our small detachments stationed at our outposts and the enemy, but as yet no wounds had been received on our side requiring the attention of surgeons.

It was soon discovered that the front of the enemy's advancing column had halted about a mile from our position, for the purpose, as was evident, of allowing time for the divisions still in the rear to arrive and take their positions in the vast line, which continued to lengthen till it had extended itself from left to right, across the valley, to the very base of the mountain, a distance of nearly a mile.

The first demonstration of the enemy against our line, was an attempt made by them to get possession of a spur of the mountain which commanded our left flank. To oppose this movement, a detachment of our riflemen was sent to occupy a like elevation to the left of our line, with orders to oppose, and if possible, to drive them from the position.

It was between these two detachments that the action commenced on the afternoon of the 22nd, and continued till dark of that day.

The wounds received, upon this first day of the battle, were mostly from spent balls, but few proving serious, and not more than two or three fatal. The extraction of a few balls, and the application of simple lint and bandaging was, therefore, so far as I know, all the surgical aid required on the evening of the 22nd.

By dawn of day on the 23d the action was again commenced between the two contending parties, both of which had kept their respective positions upon the mountains during the night; and by nine o'clock the whole enemy's force was seen advancing to attack us.

The different surgeons, with their stewards and such others as had been detailed to assist in taking care of the wounded, had already stationed themselves at convenient points near their respective regiments, ready with a plentiful supply of instruments, ligatures, bandages, splints, &c., for the arduous and responsible duties of the day.

Up to this time we had had leisure to watch the movements of the enemy, and time to indulge in some not very pleasing anticipations with regard to the result of the approaching contest. The action however soon commenced, as it seemed to us by a simultaneous discharge of musketry from both the opposing lines, and in a short time after, all thoughts upon other matters had vanished to give place to feelings of responsibility, and intense anxiety to determine correctly what to do in some cases, and what attempt in others, where to cut for a ball, and how to dress a fracture; or in case of shattered limbs, if to amputate at once, or attempt to save them.

From the hour of the attack, made upon us by the main body of the enemy in the morning, up to a time long after their retreat at night, the labor, both of body and mind, of every surgeon upon the ground, was both unremitted and constant; for it was constantly happening during the day, that long before all the cases consequent upon one charge upon the enemy could be disposed of in the most cursory and hasty manner, another desperate onset would be made to add to the number of the unfortunate still lying around us, waiting for surgical aid. Surgeons upon every part of the field were constantly being called on, amidst the din of battle, and frequently in positions as exposed as any, to attend to important cases—requiring good judgment and the best professional skill.

It would naturally be supposed that at such a time, and under such circumstances, many cases must have been entirely neglected, or if not, improperly treated. It gives me pleasure to say, on the contrary, that the opportunity which I had the day after the battle, of seeing most of the wounded and assisting in dressing such wounds as required attention, enables me to state that such cases were extremely rare.

The most common practice adopted by the different surgeons upon the field, was, in cases of gun shot wounds, to extract, if possible, all foreign substances, and in cases where balls could be felt, too far from the external wound to admit of the use of the forceps, to cut for and extract them. A simple pledget of lint, and bandage, were, in most cases all the dressings used or required. In some few cases compresses and tight bandaging were necessary to arrest hemorrhage. But few, if any cases, that I am aware of, required ligution of an artery to stop bleeding; a fact easily accounted for, when we recollect, that the divided extremities of vessels in gun-shot wounds are necessarily jagged and contused—a condition as is well known favorable to the coagulation of the blood within them, and consequently, to the prevention of hemorrhage. In cases of fractures, most of which were necessarily both compound and comminuted, the common practice was to extract all pieces of bone that were found so detached as to endanger their vitality, and to remove, as in flesh wounds, all foreign substances that could be readily found, and then to apply bandages and splints, as a temporary means of preventing motion between the fractured ends.

With regard to amputations upon the field, the rules generally adopted—were to amputate at once whenever the principal vessels and nerves of a limb had been destroyed, in cases of a fracture where the bones were found very much shattered, and in instances where important joints had been much injured.

In connection with this subject I will remark that the result of my experience, both upon the field and in the hospitals after the battle, is to convince me that the surgeon's most responsible and important duty upon the field, is to determine in the different cases when to attempt to save a limb, and when to amputate; for in a great majority of instances, so far as my knowledge extends, primary amputations were followed by favorable, and secondary by unfavorable results.

In regard to the primary treatment of gun-shot wounds, I will state, that if any mistake was committed by the surgeons, myself included, it was, in my opinion, in not being particular enough to explore thoroughly the cavities and free them from all foreign substances, such as bits of lead, cloth, paper, &c., the presence of which proved so troublesome during the subsequent treatment.

In making these remarks I do not wish to convey the impression, or express the opinion, that this apparent defect in the primary treatment was on account of carelessness or neglect on the part of myself or others; my only object in referring to the matter is to direct the attention of surgeons to the importance of devising some other means of freeing gun-shot wounds of foreign substances than those usually recommended and adopted. A simple instrument, similar to the probang used for dislodging extraneous substances from the œsophagus, for instance, might be used to cleanse all wounds admitting of its passage directly through them, excepting, perhaps, those involving important cavities.

The above remarks, hastily drawn up from memory, may serve to give our readers some conception of a surgeon's labor and responsibility upon the field. We are aware, however, that they have been altogether too general in their character to be of much professional utility, or to justify us in continuing them further at this time.

If we can hope for further indulgence from our readers, we propose to occupy a few pages in a subsequent number of the journal with a continuation of our remarks upon the after treatment, and the results of our experience in the hospitals at Saltillo.

